# A Thermoresponsive In Situ Salified Self-Nanoemulsifying Drug Delivery System of Olmesartan Medoxomil: Overcoming Leakage and Drug Trapping Limitations

**DOI:** 10.3390/pharmaceutics18091195

**Published:** 2026-09-21

**Authors:** Abdelrahman Y. Sherif, Mohammad A. Altamimi, Ehab M. Elzayat

**Affiliations:** Kayyali Research Chair for Pharmaceutical Industries, Department of Pharmaceutics, College of Pharmacy, King Saud University, Riyadh 11451, Saudi Arabia; ashreef@ksu.edu.sa (A.Y.S.); maltamimi@ksu.edu.sa (M.A.A.)

**Keywords:** self-nanoemulsifying drug delivery systems, olmesartan medoxomil, in situ salified drug loading, thermoresponsive matrix, poloxamer 188

## Abstract

**Background/Objectives:** Solid self-nanoemulsifying drug delivery systems (SNEDDS) prepared by adsorption onto porous carriers are widely used to improve the oral delivery of poorly water-soluble drugs. However, this approach is limited by increased dosing volume and incomplete drug release. This study aims to develop a thermoresponsive in situ salified SNEDDS (T-IS-SNEDDS) that simultaneously prevents formulation leakage and overcomes the release limitations of conventional adsorption-based solidification. **Methods:** Excipients were selected by solubility screening of oils and surfactants. Sodium and calcium carbonate were evaluated for in situ salt formation. Adsorption-based solid SNEDDS and thermoresponsive formulations containing poloxamer 188 with propylene glycol or polyethylene glycol 400 were prepared. Droplet size was measured after aqueous dilution. Solid-state characterization used scanning electron microscopy, Fourier transform infrared (FTIR) spectroscopy, differential scanning calorimetry, and powder X-ray diffraction. In vitro dissolution performance was evaluated against the raw drug and adsorption-based formulations. **Results:** Imwitor 308 and Tween 80 exhibited the highest solubilization capacities. Sodium carbonate (10 mg/g) markedly increased the solubility of olmesartan medoxomil from 4.11 ± 0.11 to 46.01 ± 1.92 mg/g. Although liquid in situ salified SNEDDS achieved rapid dissolution (94.2 ± 1.8% at 10 min), adsorption-based solidification required four capsules and yielded incomplete drug release (67.76 ± 3.18%). Both thermoresponsive formulations accommodated the therapeutic dose in a single capsule and released ≈95% of the drug within 60 min. **Conclusions:** The combined in situ salified drug loading and thermoresponsive matrix strategy successfully enables single capsule administration while overcoming the incomplete release of adsorption-based solid SNEDDS.

## 1. Introduction

The oral route of administration is convenient and non-invasive. These features support its widespread use and lead to exceptional patient compliance with the dosage regimen. However, the low aqueous solubility of a large fraction of therapeutic molecules limits their dissolution and oral bioavailability [1]. To tackle this limitation, self-nanoemulsifying drug delivery systems (SNEDDS) have been widely used in the literature. They consist of anhydrous blends of oil, surfactant, and cosurfactant that disperse into nanoemulsion droplets upon contact with gastrointestinal fluids under mild agitation. This improves the solubilization and absorption of lipophilic drugs [2].

Regardless of these advantages, the liquid nature of SNEDDS limits its practical market application. This is attributed to leakage from the capsule shell and to incompatibility with the capsule during storage [3]. Consequently, several techniques have been used to convert liquid SNEDDS into solid dosage forms, including spray drying, lyophilization, hot-melt extrusion, and adsorption onto porous carriers [4]. Among these, the latter has attracted particular interest owing to its simple, solvent-free, and economical single-step preparation [5].

Nevertheless, the adsorption approach has two inherent limitations that hinder its transition to large-scale pharmaceutical production. First, the low density of the porous carrier and the carrier ratio required for complete solidification increase the total volume of the solid form. Therefore, formulation often requires multiple capsules to attain the therapeutic dose of the loaded drug [6,7]. Second, a portion of the loaded formulation becomes entrapped within the carrier pores and is released incompletely [8,9]. This reduces the dose available for absorption and negatively impacts the therapeutic response. Therefore, resolving these two limitations is crucial to achieve a desirable practical single-unit solid SNEDDS.

Attaining high drug loading within liquid SNEDDS positively reduces the total volume that must be solidified and overcomes the first limitation. This limitation is particularly pronounced for weakly acidic drugs that commonly show limited solubility in SNEDDS because they remain in their unionized form [10]. Salt formation is a well-established means of raising the solubility of ionizable drugs [11]. Alkalizing agents have been used for this purpose in various dosage forms [12,13]. In those systems, they increase drug solubility by raising the microenvironmental pH surrounding the drug.

Therefore, adding sodium carbonate to liquid SNEDDS promotes proton transfer from the ionizable drug to the base. It converts the drug into a more soluble salt and increases drug loading. A comparable gain in lipid vehicle loading was previously attained using preformed lipophilic salts [14]. Consequently, the resulting formulation is referred to as the liquid in situ salified SNEDDS (L-IS-SNEDDS).

However, incomplete release from the porous carrier requires an alternative solidification strategy to fully liberate the formulation in vivo. In this regard, a thermoresponsive matrix that remains solid during storage and reverts to a liquid at body temperature offers such a solution. This behavior can be achieved using a poloxamer dissolved in nonaqueous solvents such as propylene glycol and polyethylene glycol 400.

In aqueous media, poloxamers assemble into micelles that pack into ordered structures and solidify upon heating [15]. This behavior could be inverted when water is replaced by a nonaqueous solvent. Reversed micellar structures with a poly(ethylene oxide) core have been reported in ethanol-rich media, and their ordered domains melt upon heating [16]. Similarly, Pluronic F-127 dissolved in a glycol solvent remained fluid on heating and solidified only upon cooling. Hydrogen bonding, rather than the lyotropic packing that governs aqueous systems, was considered responsible for this behavior [17].

Consequently, combining poloxamer with a nonaqueous solvent allows tuning of this transition and yields thermoresponsive SNEDDS. Complete liquefaction of the matrix agents ensures complete dispersion of the SNEDDS formulation and the loaded agent, without the trapping risk seen in adsorption. Incorporating the salt-loaded formulation into this matrix yields the final optimized system: the thermoresponsive in situ salified SNEDDS (T-IS-SNEDDS).

Olmesartan medoxomil was selected as a model drug to evaluate this combined strategy. It is an orally administered angiotensin II receptor blocker used to treat high blood pressure. However, the lipophilic nature of olmesartan medoxomil results in an absolute oral bioavailability of only 26% [18]. Olmesartan medoxomil is highly lipophilic (log P = 4.31) and carries a weakly acidic tetrazole group (pK_a_ ≈ 4.3) [19]. These properties make it suitable for both solubilization within a lipid vehicle and ionization-based loading enhancement.

Therefore, this study aimed to develop a formulation that combines thermoresponsive behavior with in situ salified drug loading. This could simultaneously overcome the drug-loading and incomplete-release limitations of conventional adsorption-based solid SNEDDS. The oil and surfactant were first selected from a solubility screening of olmesartan medoxomil. Next, two carbonate alkalizing agents (sodium carbonate and calcium carbonate) were used to assess their impact on drug loading. The proposed poloxamer 188 matrices, containing either propylene glycol or polyethylene glycol 400, were characterized by Fourier transform infrared spectroscopy, differential scanning calorimetry, and powder X-ray diffraction. The L-IS-SNEDDS was subsequently solidified using poloxamer-based systems with two types of nonaqueous solvents. For comparative assessment, a traditional solid form was prepared using Syloid as a solidifying agent. The self-emulsification behavior and droplet size of all formulations were assessed after aqueous dilution. The aforementioned formulations were evaluated for in vitro dissolution against the raw drug and the conventional Syloid adsorbate.

## 2. Materials and Methods

### 2.1. Materials

Riyadh Pharma (Riyadh, Saudi Arabia) kindly donated olmesartan medoxomil. The oils used for solubility screening were obtained as follows: oleic acid from Avonchem Ltd. (Macclesfield, Cheshire, UK); Imwitor 308 and Miglyol 810N from Sasol Germany GmbH (Witten, Germany); Peceol and Maisine 35-1 from Gattefossé (Saint-Priest, France); Captex 355 from Abitec Corporation (Janesville, WI, USA); soybean oil from John L. Seaton & Co., Ltd. and Croda International Plc. (Hull, UK); and arachis oil from Winlab Laboratory Chemicals (Market Harborough, UK). The surfactants Tween 60 and Tween 85 were supplied by Merck-Schuchardt OHG (Hohenbrunn, Germany), Tween 20 by BDH (Poole, UK), and Tween 80 by Techno Pharmchem (Bahadurgarh, India). The PEG-hydrogenated castor oil surfactants HCO-60, HCO-30, and HCO-10 were obtained from Nikko Chemicals Co., Ltd. (Tokyo, Japan). The nonaqueous matrix solvents propylene glycol and polyethylene glycol 400 (Kollisolv PEG 400) were obtained from Winlab Laboratory Chemicals (Market Harborough, UK) and BASF (Ludwigshafen, Germany), respectively. Syloid was purchased from W. R. Grace & Co. (Columbia, MD, USA).

### 2.2. Solubility Study

An excess amount of olmesartan medoxomil was added to each tested agent (oils; Imwitor 308/surfactant mixtures, 1:1 *w*/*w*; and Imwitor 308/Tween 80 (1:1 *w*/*w*) containing sodium carbonate or calcium carbonate at 2–10 mg/g concentrations) in a 10 mL glass beaker and stirred at 800 rpm on a five-position magnetic stirrer (RO 5, IKA-Werke, Staufen, Germany) for 24 h at ambient laboratory temperature (23.0 ± 2.0 °C). Each mixture was transferred to a 2.0 mL microcentrifuge tube (Expell Secure, CAPP, Denmark) and centrifuged at 13,000 rpm for 20 min in a MIKRO 20 microcentrifuge fitted with a 24-place fixed-angle rotor (Andreas Hettich GmbH & Co. KG, Tuttlingen, Germany). The clear supernatant was withdrawn into a fresh tube to prevent redispersion of the sediment during handling. A single droplet of the supernatant was dispensed with a micropipette into a new microcentrifuge tube on an analytical balance (HR-202i, A&D Company Ltd., Tokyo, Japan; readability 0.1 mg), and its mass was recorded. Acetonitrile (1.8 mL) was added, and the sample was sonicated for 15 min in an ultrasonic bath (375H, Jencons (Scientific) Ltd., Leighton Buzzard, UK) at ambient temperature. Samples were diluted further as required to fall within the calibration range, giving overall dilution factors of 1–20 depending on the agent.

### 2.3. Preparation of Liquid SNEDDS (L-SNEDDS)

The L-SNEDDS was prepared by blending Imwitor 308 as the oil phase and Tween 80 as the surfactant at a 1:1 (*w*/*w*) ratio. Both excipients were shaken and placed in an incubator set to 40 °C for at least 15 min until a clear and homogeneous mixture was obtained.

### 2.4. Preparation of Liquid In Situ Salified SNEDDS (L-IS-SNEDDS)

L-IS-SNEDDS was prepared by incorporating sodium carbonate (10 mg/g) into the L-SNEDDS, followed by dispersion under magnetic stirring. Olmesartan medoxomil was added to prepare drug-loaded L-IS-SNEDDS at a loading of 20 mg/g, and the mixture was stirred until a clear solution was obtained.

### 2.5. Preparation of Solid In Situ Salified SNEDDS by Adsorption (S-IS-SNEDDS)

The conventional solid form was prepared by adsorbing the drug-loaded L-IS-SNEDDS onto Syloid at a 1:1 (formulation: adsorbent, *w*/*w*) ratio. The two components were gently mixed until a uniform, free-flowing powder was obtained, yielding the solid in situ salified SNEDDS (S-IS-SNEDDS) used as the adsorption-based comparator.

### 2.6. Preparation of Thermoresponsive In Situ Salified SNEDDS (T-IS-SNEDDS)

The thermoresponsive matrix-based formulations were prepared using a matrix former consisting of poloxamer 188 (solidifying polymer) and a nonaqueous solvent (propylene glycol or polyethylene glycol 400) that dissolves the polymer. Poloxamer 188 and the nonaqueous solvent were added to the drug-loaded L-IS-SNEDDS at 20% *w*/*w* each (60% *w*/*w* L-IS-SNEDDS), and the mixture was heated above 40 °C until complete dissolution of the poloxamer. For mechanistic characterization, binary matrices of poloxamer 188 and the respective solvent (20% *w*/*w* poloxamer 188) were prepared in the same manner, without L-IS-SNEDDS.

### 2.7. Self-Emulsification and Droplet Size Analysis

The drug-loaded L-IS-SNEDDS, S-IS-SNEDDS, and the propylene glycol- and polyethylene glycol 400-based T-IS-SNEDDS were dispersed in purified water pre-warmed to 37 ± 0.5 °C at a formulation-to-water ratio of 1:500 (*w*/*v*). The S-IS-SNEDDS dispersions were centrifuged to sediment the silica from the supernatant. The mean droplet size and polydispersity index of the resulting dispersions were measured by dynamic light scattering using a Zetasizer (ZEN3600, Malvern Instruments, Malvern, UK) at 25 °C.

### 2.8. Scanning Electron Microscopy (SEM)

We investigated the surface morphology of raw olmesartan medoxomil, Syloid, and the drug-loaded S-IS-SNEDDS using scanning electron microscopy (Carl Zeiss EVO LS10, Cambridge, UK). For SEM analysis, the powder samples were mounted on aluminum stubs and sputter-coated with a thin gold layer. The coated samples were then scanned using a scanning electron microscope equipped with a secondary electron detector (SE1) at an accelerating voltage of 5.00 kV to obtain comprehensive information on the particles’ surface morphology.

### 2.9. Fourier Transform Infrared (FTIR) Spectroscopy

The molecular interactions governing the thermoresponsive solid-to-liquid transition were investigated by FTIR using a PerkinElmer Spectrum-100 spectrometer (PerkinElmer Inc., Waltham, MA, USA) fitted with a diamond attenuated total reflectance accessory. Raw olmesartan medoxomil, sodium carbonate, Syloid, poloxamer 188, propylene glycol, polyethylene glycol 400, the drug-free L-SNEDDS, the drug-free and drug-loaded L-IS-SNEDDS, and the drug-loaded S-IS-SNEDDS were scanned at ambient temperature over 4000–650 cm^−1^ (resolution 4 cm^−1^). The binary matrices and the drug-loaded T-IS-SNEDDS were additionally recorded in the solid (ambient) and liquid (50 °C) states to track the crystalline poloxamer markers across the thermal transition. Spectra were acquired and processed using Spectrum software version 6.3.5 (PerkinElmer Inc., Waltham, MA, USA).

### 2.10. Differential Scanning Calorimetry (DSC)

Thermal analysis of olmesartan medoxomil, poloxamer 188, Syloid, the binary poloxamer 188/propylene glycol and poloxamer 188/polyethylene glycol 400 matrices, the drug-loaded S-IS-SNEDDS, and the drug-loaded propylene glycol- and polyethylene glycol 400-based T-IS-SNEDDS was achieved utilizing a DSC-4000 PerkinElmer (Waltham, MA, USA) apparatus. Agents were accurately weighed into aluminum pans and sealed. Thermograms for analyzed samples were recorded at a heating rate of 5 °C/min within the predetermined range of 30–250 °C, whereas the binary matrices were scanned within 30–70 °C to capture the matrix transition.

### 2.11. Powder X-Ray Diffraction (XRD)

X-ray diffraction was used to investigate the crystalline state of olmesartan medoxomil and poloxamer 188 in the tested agents. Olmesartan medoxomil, poloxamer 188, Syloid, the binary poloxamer 188/propylene glycol and poloxamer 188/polyethylene glycol 400 matrices, the drug-loaded S-IS-SNEDDS, and the drug-loaded propylene glycol- and polyethylene glycol 400-based T-IS-SNEDDS were scanned using an X-ray diffractometer (Ultima IV, Rigaku Inc., Tokyo, Japan) equipped with Cu Kα radiation (40 kV, 30 mA), a scintillation counter, and a fixed monochromator. Samples were loaded in the position and spun at 1 rpm during measurement. The diffraction patterns were acquired in 2θ/θ continuous-scan mode at 1.000°/min with a sampling width of 0.1° over the angular range 3–30° (2θ), using 2/3° divergence and scatter slits, a 10 mm height-limiting slit, and a 0.3 mm receiving slit. Data processing included Savitzky–Golay smoothing, Sonneveld–Visser background subtraction, and Rachinger Kα_2_ elimination.

### 2.12. In Vitro Dissolution Study

In vitro dissolution was performed using a USP Type II (paddle) apparatus (LOGAN Inst. Corp., Somerset, NJ, USA). Hard gelatin capsules were filled with raw olmesartan medoxomil or with the test formulations (L-IS-SNEDDS, S-IS-SNEDDS, and the propylene glycol-based and polyethylene glycol 400-based T-IS-SNEDDS) containing an equivalent of 20 mg of olmesartan medoxomil, and each capsule was enclosed in a sinker to prevent floating. The dissolution medium consisted of 900 mL of phosphate buffer (pH 6.8) maintained at 37 ± 0.5 °C with the paddle rotating at 50 rpm. Samples were withdrawn at 5, 10, 15, 30, 45, and 60 min through a syringe fitted with a 10 µm filter. All experiments were performed in triplicate. The dissolution efficiency (DE) was calculated as the area under the dissolution curve up to 60 min expressed as a percentage of the area of the rectangle describing 100% dissolution over the same period. The initial dissolution rate was calculated as the percentage dissolved at 5 min divided by 5 min.

## 3. Results and Discussion

**Figure 1:**
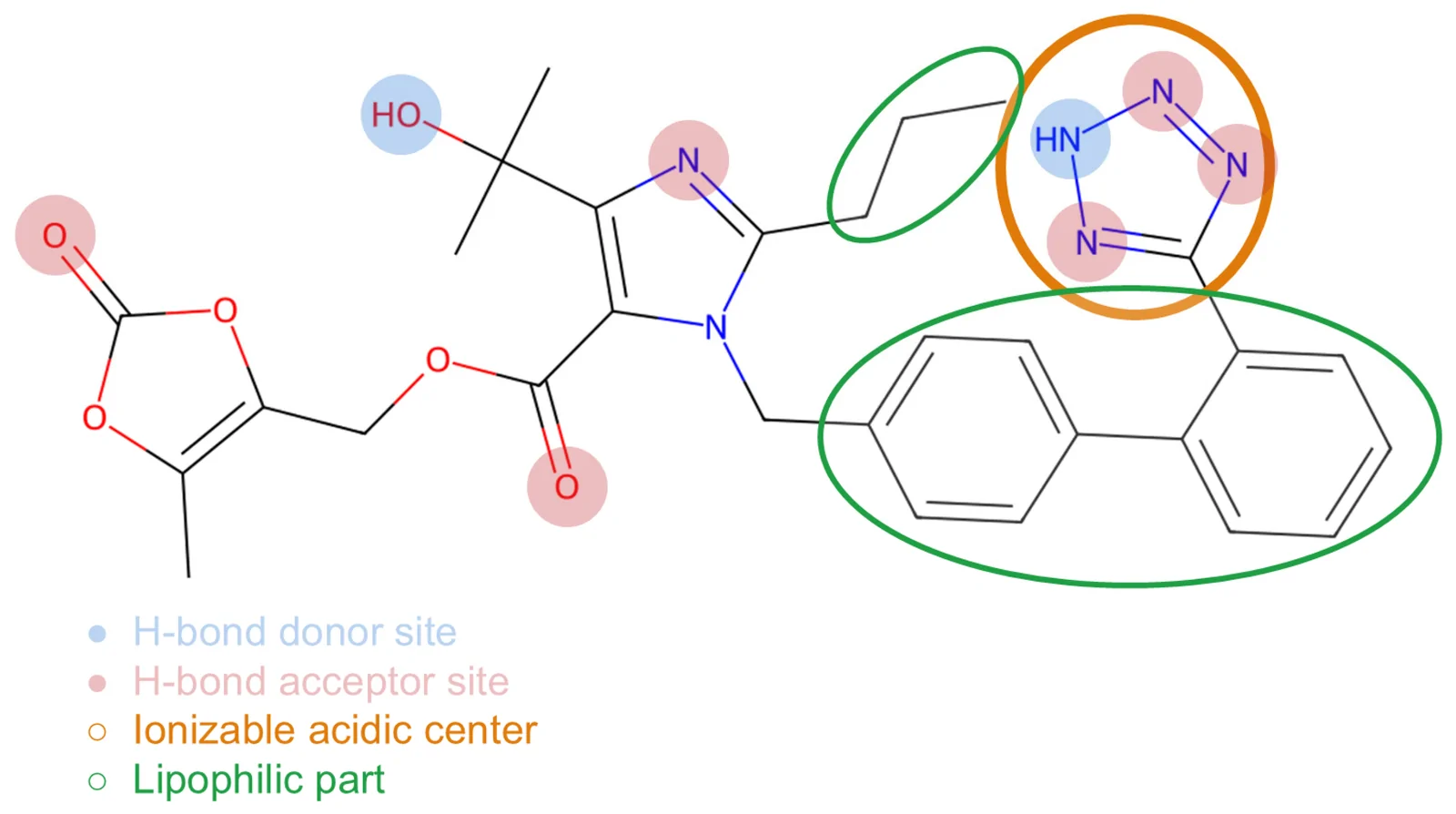
Structural features of olmesartan medoxomil: hydrogen-bond donors (light blue), hydrogen-bond acceptors (light red), the ionizable tetrazole (orange circle), and the lipophilic framework (green circle).

The solubility of olmesartan medoxomil is controlled by its molecular structure. Figure 1 shows the chemical structure of olmesartan medoxomil, with the potential binding sites marked. It contains a tertiary hydroxyl group, ester and carbonyl oxygens, an imidazole nitrogen, and a tetrazole ring. The hydroxyl and the tetrazole act as hydrogen-bond donors, while the carbonyl and nitrogen atoms act as acceptors. Therefore, its solubility depends on how effectively an excipient engages these groups through hydrogen bonding. In contrast, the high lipophilicity of olmesartan medoxomil is attributed to two hydrophobic moieties. These are the biphenyl system and the n-propyl chain, which is consistent with its reported log P of 4.31 [19]. From another perspective, the tetrazole N–H ionizable group accounts for its acidic character. Consequently, its solubilization could be enhanced through salt formation and dissolution in the system’s hydrophilic components.

In this context, the solubility of olmesartan medoxomil in SNEDDS determines the final total dosage volume and affects its pharmaceutical applicability. The solubilization of olmesartan medoxomil is influenced by opposing factors related to its chemical structure. Thus, this study screened excipients (oil, surfactant, and salts) with different chemical structures to identify the dominant factor governing loading in the SNEDDS formulation. This helps select formulation excipients that achieve therapeutic drug loading with less formulation volume.

### 3.1. Solubility in Oils

The measured solubility of olmesartan medoxomil within various kinds of oils is presented in Table 1. The results showed that olmesartan medoxomil exhibited limited solubility (<0.1 mg/g) in oils comprising triglycerides (Miglyol 810N, Captex 355, soybean oil, and arachis oil) and free fatty acid (oleic acid). In contrast, oils composed of monoglycerides (Imwitor 308, peceol, and maisine 35-1) showed markedly higher solubility, with the highest solubility in Imwitor 308.

The negligible solubility of olmesartan medoxomil in triglycerides could be attributed to the complete esterification of all three hydroxyl groups of the glycerol backbone with fatty acid chains [20]. The hydrophobic microenvironment of the aforementioned oils reduces their ability to solubilize olmesartan medoxomil, which contains multiple polar functional groups. Consequently, the observed low solubility could be ascribed to the limited olmesartan medoxomil–excipient interactions. Similarly, the low solubility of olmesartan medoxomil in oleic acid can be ascribed to the unavailability of its single carboxylic acid group. This group tends to self-associate into cyclic hydrogen-bonded dimers in neat and apolar lipids [21,22]. This limits its availability to hydrogen-bond with the polar functional groups of olmesartan medoxomil.

On the other hand, olmesartan medoxomil exhibited higher solubility in monoglycerides enriched with two free hydroxyl groups [20]. This agrees with previously published studies showing that olmesartan medoxomil has a strong tendency to form hydrogen bonds with various agents [19,23,24]. Nevertheless, the observed highest drug solubility in Imwitor 308, compared with other monoglycerides, is due to its lower molecular weight. This gives a higher hydroxyl density per unit mass. This is consistent with reported quantitative relationships in which solute solubility in glyceride media correlates with the molar concentration of hydroxyl groups [25].

Imwitor 308 was chosen as the oil phase based on a solubility study, offering numerous advantages from various perspectives. Firstly, it reduces the total volume of formulation to be administered and increases patient compliance [26]. In addition, it raises the drug’s association opportunity within the core of the nanoemulsion system. This enhances its potential for solubilization following oral administration in the gastrointestinal tract [27]. Finally, maximizing solubilization in the gastrointestinal lumen promotes the movement of drug molecules towards systemic circulation via a concentration gradient [28].

### 3.2. Solubility in Surfactants

The solubility of olmesartan medoxomil in various surfactants, evaluated in the context of an Imwitor 308-based SNEDDS formulation, is presented in Table 2. Among all tested surfactants, the formulation containing Tween 80 demonstrated the highest solubilization capacity (4.11 ± 0.11 mg/g), followed by Tween 20 (2.73 ± 0.07 mg/g), HCO-60 (2.68 ± 0.19 mg/g), Tween 60 (2.46 ± 0.02 mg/g), HCO-30 (2.30 ± 0.04 mg/g), and Tween 85 (2.05 ± 0.13 mg/g), while HCO-10 showed the lowest solubility (1.34 ± 0.07 mg/g).

The current results align with the observed results of the oil phase. In this context, the solubilization of olmesartan medoxomil within the anhydrous lipid–surfactant matrix appears to be governed predominantly by hydrogen bonding. This occurs between the drug’s polar functional groups and the oxygen functionalities of the excipient. Consequently, any structural factor that renders these hydrogen-bonding interactions more accessible or achievable favors higher solubility [18,29]. This finding is clearly observed in polysorbates, which carry a comparable sorbitan–polyoxyethylene head group and differ mainly in the fatty acyl tail type. The fatty acyl tail nature modulates olmesartan medoxomil’s access to the hydrogen-bonding region. For example, the superior performance of the formulation containing Tween 80 over those containing Tween 20 and 60 could be attributed to the monounsaturated oleate chain. Its cis double bond introduces a permanent kink that loosens molecular packing. This creates a more fluid domain through which the bulky olmesartan medoxomil molecule can reach the ether oxygens [30,31]. On the other hand, the saturated fatty acyl tails of Tween 60 (stearate) and Tween 20 (laurate) form a tightly packed domain [31,32]. This restricts the olmesartan medoxomil’s access to the bonding sites and lowers its solubility. In contrast, Tween 85 was the least effective of the polysorbates. This could be ascribed to its predominantly triester structure, in which three esterified oleate chains crowd and sterically shield the sorbitan–polyoxyethylene head. Therefore, the highest solubility of the formulation containing Tween 80 could be ascribed to the greater accessibility of its hydrogen-bond-accepting ether oxygens. Because the number of polyoxyethylene units is comparable among the polysorbates, this accessibility arises from the loosely packed monounsaturated oleate chain rather than from a greater abundance of ether oxygens.

The solubility of olmesartan medoxomil in PEG–hydrogenated castor oil (HCO) surfactant diminished as the degree of ethoxylation decreased. The obtained results showed a decrease in olmesartan medoxomil solubility from HCO-60 (2.68 ± 0.19 mg/g) to HCO-30 (2.30 ± 0.04 mg/g), then to HCO-10 (1.34 ± 0.07 mg/g). This finding aligns with the principle that these surfactants share a lipophilic backbone and differ only in their degree of ethoxylation. Therefore, the difference in the number of ether oxygens available to form hydrogen bonds with olmesartan medoxomil led to differences in drug solubility [33,34]. For example, HCO-60 has the most polyoxyethylene units and therefore offers the most hydrogen-bonding sites. This resulted in the highest drug solubility in PEG–hydrogenated castor oil surfactants. On the other hand, the reduction in polyoxyethylene units in HCO-10 resulted in the lowest solubility of olmesartan medoxomil [35]. Overall, it could be concluded that the solubility of olmesartan medoxomil depends mainly on the number of accessible hydrogen-bonding sites with the excipients in the formulation. Since Tween 80 exhibited the highest solubility among the surfactants tested (4.11 mg/g), it was selected to achieve the highest possible drug loading within the formulation.

### 3.3. Solubility in In Situ Salified SNEDDS

The measured solubility of olmesartan medoxomil within the selected SNEDDS composed of Tween 80 and Imwitor 308 was 4.11 ± 0.11 mg/g. This value is insufficient to accommodate the required therapeutic loading within a single dosage unit. The solubilization capacity of a self-nanoemulsifying system is governed primarily by the physicochemical interactions between the drug and its lipid and surfactant components [29]. Even after screening a wide range of excipients with diverse chemical structures, the intrinsic solubility of olmesartan medoxomil in anhydrous vehicles limits achieving the required drug loading [14]. In this regard, the drug’s ionization behavior was exploited as an alternative approach to achieve this goal. In olmesartan medoxomil, the weakly acidic tetrazole N–H (pK_a_ ≈ 4.3) is the principal ionizable group available for deprotonation by a base [36,37]. Furthermore, olmesartan medoxomil is practically insoluble between pH 3.5 and 6. Consequently, we incorporated sodium carbonate and calcium carbonate at 2–10 mg/g to deprotonate the drug and enhance solubility through in situ salt formation. Table 3 summarizes the solubility of olmesartan medoxomil within the SNEDDS containing sodium carbonate and calcium carbonate across this loading range.

#### 3.3.1. Sodium Carbonate

Sodium carbonate produced a concentration-dependent increase in drug solubility. The solubility increased from 17.78 ± 0.62 mg/g at 2 mg/g to 46.01 ± 1.92 mg/g at 10 mg/g. This enhancement can be attributed to in situ salt formation in an anhydrous SNEDDS formulation. Sodium carbonate is freely soluble in water and has strong alkaline properties. Therefore, it serves as a strong base for abstracting the proton from the weakly acidic tetrazole. The resulting sodium salt of the drug is considerably more soluble than the parent acid form of olmesartan medoxomil. Comparable gains have been reported when poorly soluble compounds are converted to lipophilic salts or ionic liquids before incorporation into lipid vehicles, with solubility increases of approximately 2- to 80-fold [14,38]. In situ salt formation during manufacture has likewise been applied to amorphous solid dispersions [39]. In the SNEDDS formulation, the olmesartan medoxomil salt could be associated with the polar microdomains (ethoxylate and glyceryl-hydroxyl).

#### 3.3.2. Calcium Carbonate

In contrast, calcium carbonate produced only a marginal (≈1.4-fold), concentration-independent increase in olmesartan medoxomil solubility. The current results showed that drug solubility remained at approximately 5.7 mg/g over the 2–10 mg/g range. This observation can be attributed to the differing solubilities of the two types of carbonate salts in the formulation. Sodium carbonate is freely soluble in water, whereas calcium carbonate is practically insoluble. Consequently, calcium carbonate cannot dissolve to abstract the tetrazole proton, and salt formation cannot occur. The counterion behavior further supports this outcome. Divalent calcium salts of organic acids are generally far less soluble than their monovalent sodium counterparts. This is attributed to stronger electrostatic attraction within the crystal lattice.

### 3.4. Assessment of In Situ Salt Formation

**Figure 2:**
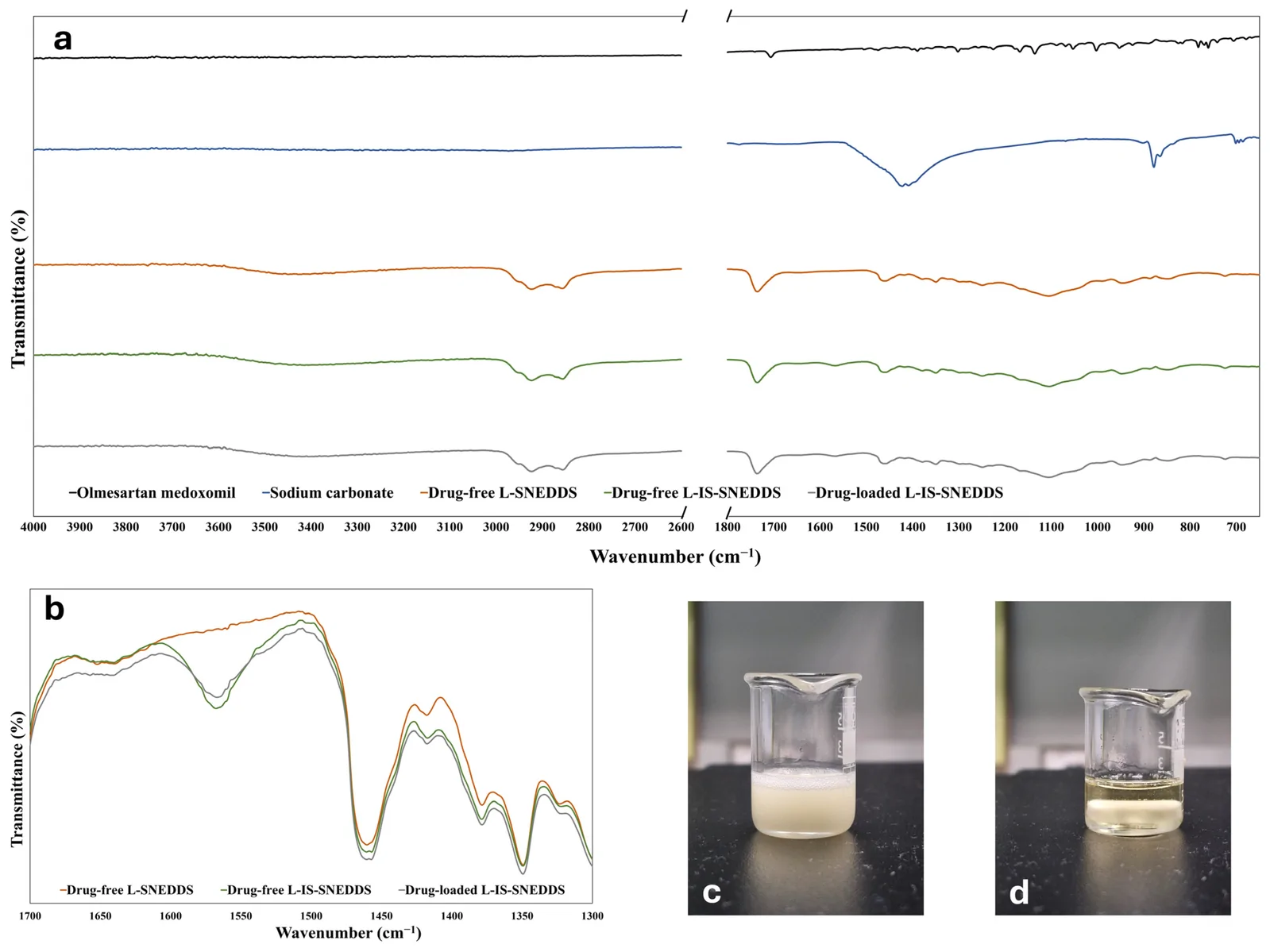
(**a**) FTIR spectra of raw olmesartan medoxomil, sodium carbonate, the drug-free L-SNEDDS, the drug-free L-IS-SNEDDS, and the drug-loaded L-IS-SNEDDS. Spectra are offset vertically for clarity. (**b**) Expansion of the 1700–1300 cm^−1^ region. Physical appearance of (**c**) the L-SNEDDS and (**d**) the L-IS-SNEDDS after addition of olmesartan medoxomil.

The formation of the olmesartan medoxomil sodium salt was examined by FTIR spectroscopy. Spectra of the raw drug, sodium carbonate, the drug-free L-SNEDDS, the drug-free L-IS-SNEDDS, and the drug-loaded L-IS-SNEDDS are presented in Figure 2. The three formulations (drug-free L-SNEDDS, drug-free L-IS-SNEDDS, and drug-loaded L-IS-SNEDDS) have similar spectra, except for two features. In contrast to drug-free L-SNEDDS, the two carbonate-containing formulations (drug-free L-IS-SNEDDS and drug-loaded L-IS-SNEDDS) have a band at 1567 cm^−1^ and a shoulder near 1404 cm^−1^. These could correspond to sodium salts of fatty acids [40,41]. The observation of both peaks in drug-free L-IS-SNEDDS rules out olmesartan medoxomil as their source. Therefore, it is expected that the presence of free fatty acids in the used oil (Imwitor 308) and the surfactant (Tween 80) are the main contributors to such observation. Moreover, the intensity of the peak at 1567 cm^−1^ was weaker in the drug-loaded formulation compared to the drug-free one. This suggests that olmesartan medoxomil consumed part of the added sodium carbonate. Thus, it reduced the amount of fatty acid salt formed and the intensity of its band. Moreover, the methylene band near 723 cm^−1^ was unchanged and remained a single peak. This indicates that the salt remains dispersed in the liquid rather than separating as a solid [42]. Figure 2c,d show the physical appearance of the L-SNEDDS and L-IS-SNEDDS formulations after mixing with olmesartan medoxomil. It is clear that olmesartan medoxomil was completely dissolved in L-IS-SNEDDS, as indicated by the transparent appearance of the formulation. This confirms that sodium carbonate is the main contributor to olmesartan medoxomil solubilization in L-IS-SNEDDS.

### 3.5. Pharmaceutical Assessment of L-IS-SNEDDS and S-IS-SNEDDS

**Figure 3:**
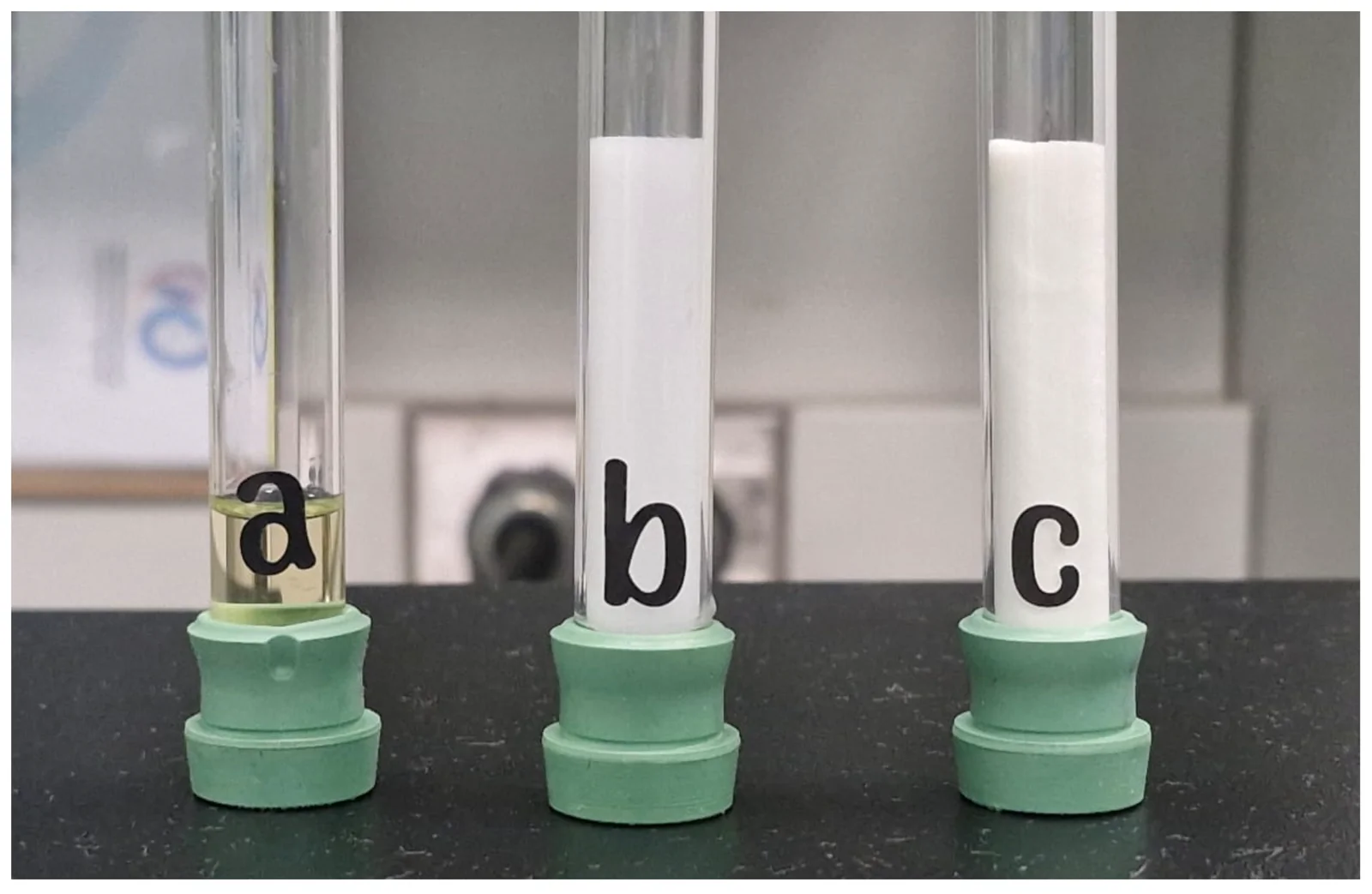
Visual comparison of the volume occupied by (**a**) 1 g of the drug-loaded L-IS-SNEDDS, (**b**) 1 g of the Syloid adsorbent, and (**c**) the drug-loaded S-IS-SNEDDS prepared from 1 g of each (2 g).

Figure 3 shows the impact of adsorption-based solidification on total dosage volume. One gram of Syloid occupies approximately four times the volume of 1 g of drug-loaded L-IS-SNEDDS. This is ascribed to the low bulk density and porous nature of the Syloid carrier. The prepared drug-loaded S-IS-SNEDDS (2 g) occupies the same volume as 1 g of the Syloid carrier. This indicates successful adsorption of drug-loaded L-IS-SNEDDS within the porous structure of Syloid. Moreover, the self-emulsification behavior of the prepared formulations after aqueous dilution was evaluated. The L-IS-SNEDDS spontaneously dispersed into nanosized droplets (214.3 ± 4.9 nm) with a polydispersity index of 0.406 ± 0.025. Solidification by adsorption onto Syloid produced nanoemulsion droplets (223.5 ± 6.0 nm) with a polydispersity index of 0.702 ± 0.119. The prepared drug-loaded L-IS-SNEDDS and S-IS-SNEDDS were exposed to in vitro dissolution assessment.

**Figure 4:**
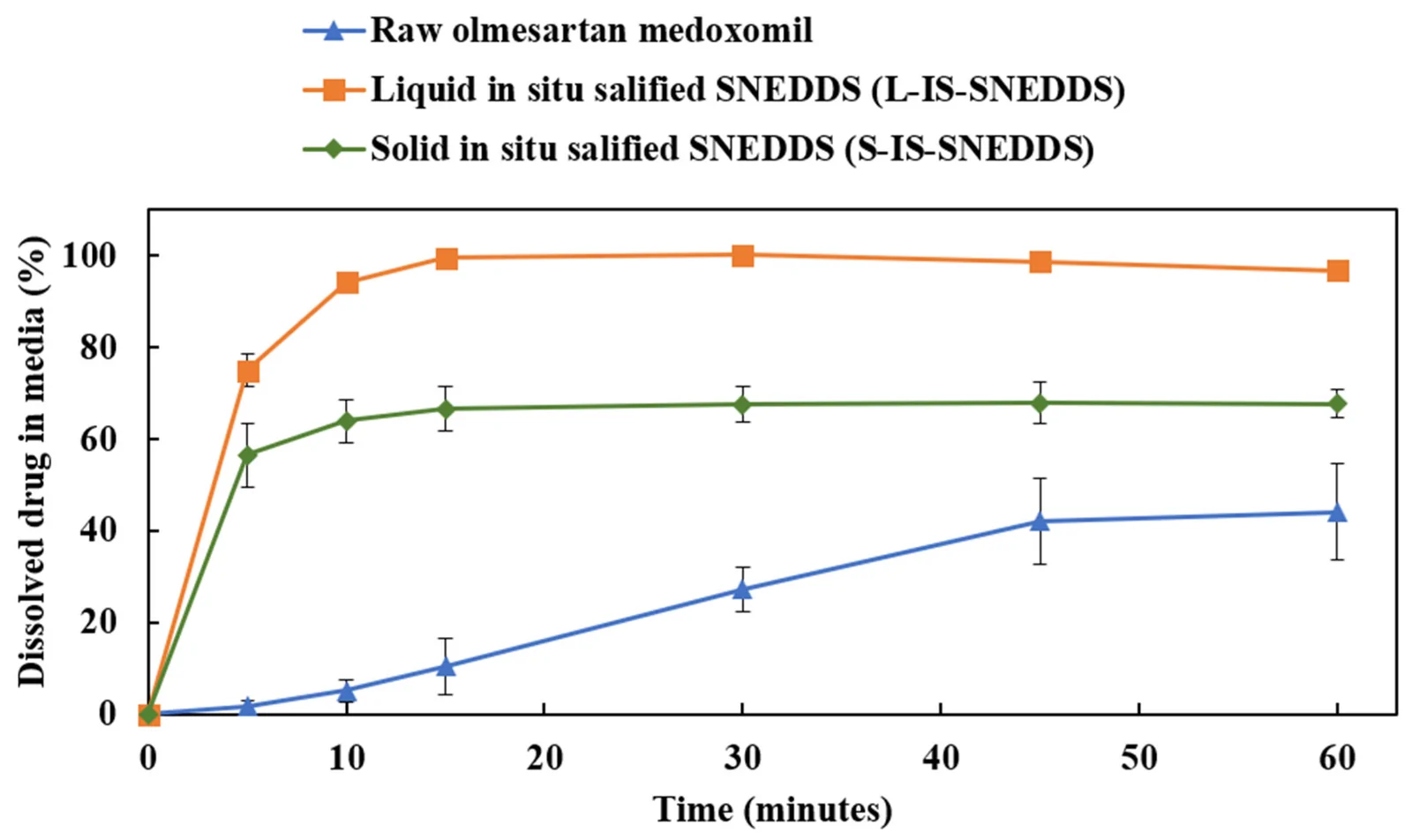
In vitro dissolution of raw olmesartan medoxomil, liquid in situ salified SNEDDS (L-IS-SNEDDS), and solid in situ salified SNEDDS (S-IS-SNEDDS).

Building on the enhanced drug loading achieved by adding sodium carbonate, we evaluated the dissolution behavior of the in situ salified SNEDDS in liquid form and after solidification onto the porous adsorbent. The in vitro dissolution profiles of raw olmesartan medoxomil, liquid in situ salified SNEDDS (L-IS-SNEDDS), and solid in situ salified SNEDDS (S-IS-SNEDDS) are presented in Figure 4.

#### 3.5.1. Liquid Formulation

The current results showed that L-IS-SNEDDS enhanced the dissolution profile of olmesartan medoxomil, with 94.2 ± 1.8% dissolved at 10 min compared to the raw drug (5.2 ± 2.5%). Moreover, olmesartan medoxomil dissolution efficiency from L-IS-SNEDDS was 92.5%, with an initial dissolution rate of 15.0%/min [43]. Three mechanisms account for this superior performance of L-IS-SNEDDS. First, the formulation presents the drug in a pre-dissolved state. This bypasses the dissolution-rate-limiting step of dissolving the drug from the crystalline powder. Second, in situ salt formation with sodium carbonate produces a water-soluble salt form of olmesartan medoxomil that is ready for dissolution and reduces the driving force for precipitation. Third, L-IS-SNEDDS spontaneously emulsifies upon contact with the medium and produces nanosized droplets of 214.3 ± 4.9 nm.

#### 3.5.2. Solid Formulation

Leakage and incompatibility with the capsule shell limit the pharmaceutical applicability of L-SNEDDS. Therefore, a solid SNEDDS was prepared using adsorption technology to overcome the aforementioned limitation. The prepared S-IS-SNEDDS required four capsules per dose because the Syloid adsorbent has a low bulk density (Figure 3). In addition, only 67.76 ± 3.18% of the drug was released from the adsorbent matrix at the end of the experiment. The plateau observed from 10 to 60 min provides indirect evidence of drug trapping within the porous structure of the Syloid adsorbent. The calculated dissolution efficiency from S-IS-SNEDDS was only 63.5%. This finding is consistent with previously reported studies that showed drug entrapment within the porous structure of the Syloid carrier [44]. It also aligns with the reported penetration of the SNEDDS formulation within the mesoporous structure of the silica adsorbent. The nanoscale confinement within the silica is too restrictive to permit full emulsification of the SNEDDS [45]. Therefore, a fraction of SNEDDS could be trapped within the mesopores, resulting in the incomplete release plateau observed in the present data.

### 3.6. Solid-State Characterization of the Adsorption-Based System

**Figure 5:**
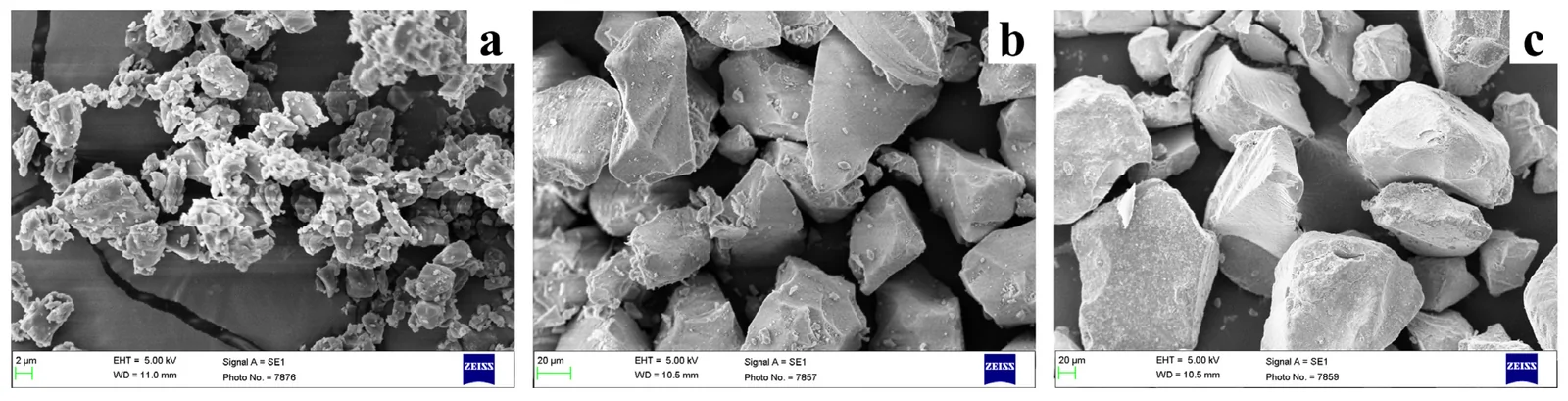
Scanning electron micrographs of (**a**) raw olmesartan medoxomil, (**b**) the Syloid adsorbent, and (**c**) the drug-loaded S-IS-SNEDDS.

Although the L-IS-SNEDDS formulation addresses the limited drug-loading capacity of L-SNEDDS, its leakage potential still needs to be addressed. Therefore, the S-IS-SNEDDS formulation was prepared using Syloid adsorbent to reduce leakage. The prepared S-IS-SNEDDS formulation was subjected to physical-state characterization to assess how this method affected the drug’s physical state after solidification. Figure 5 shows the SEM images of olmesartan medoxomil, the adsorbent, and the drug-loaded S-IS-SNEDDS. Figure 5a shows that olmesartan medoxomil has an irregular crystalline shape consistent with a previously reported study [46]. Moreover, the adsorption of drug-loaded L-IS-SNEDDS on Syloid was achieved without any observation of crystalline drug. In addition, the retention of the Syloid adsorption structure indicates that L-IS-SNEDDS is present inside its pores. However, further investigation was carried out to check the physical state of the olmesartan medoxomil within the core of Syloid.

**Figure 6:**
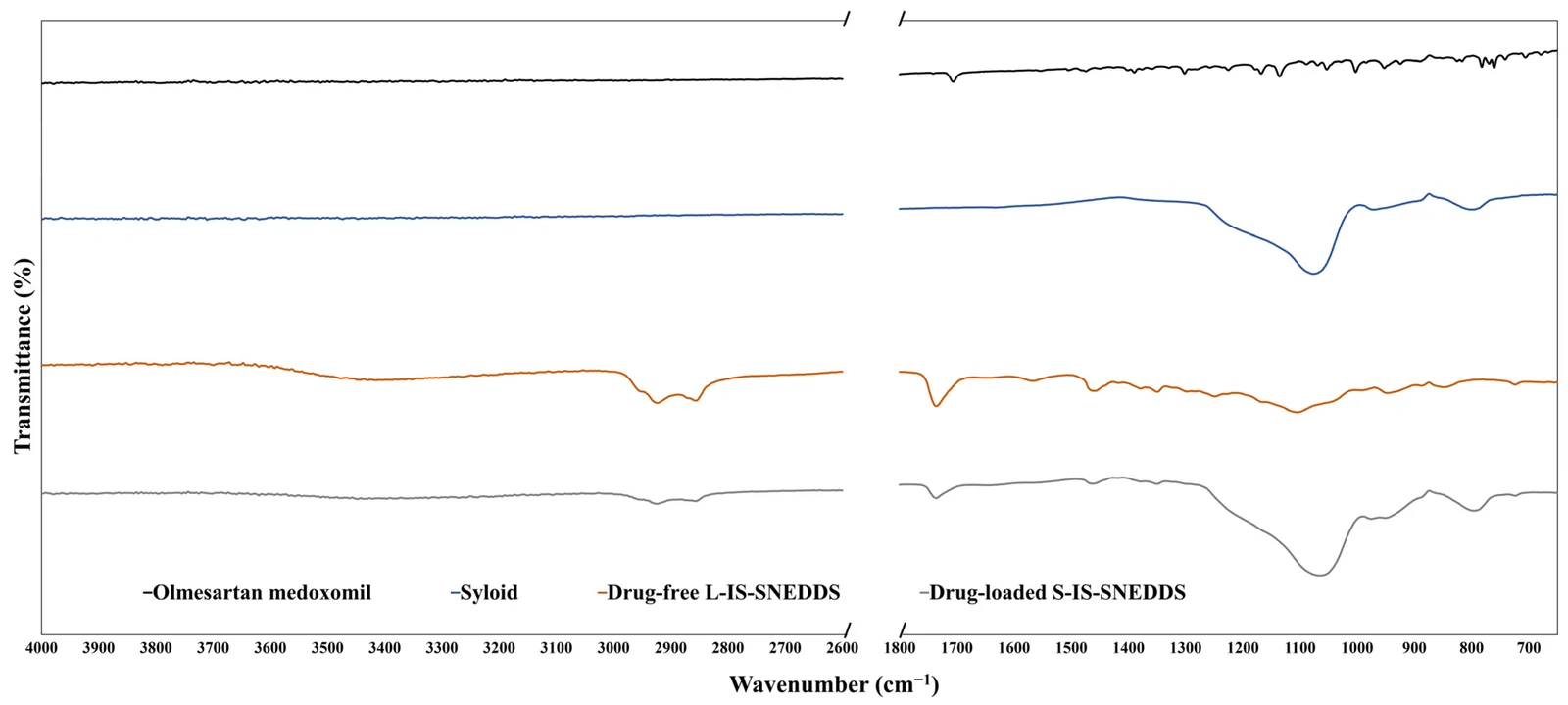
FTIR spectra of raw olmesartan medoxomil, Syloid, the drug-free L-IS-SNEDDS, and the drug-loaded S-IS-SNEDDS.

The infrared spectrum of Syloid (Figure 6) showed the asymmetric Si–O–Si stretching peak between 1000 and 1200 cm^−1^, together with the associated silanol band near 960 cm^−1^ and the symmetric Si–O–Si band near 800 cm^−1^. The spectra of the drug-loaded S-IS-SNEDDS corresponded to a straightforward superposition of the silica and L-IS-SNEDDS spectra. Moreover, no new bands and no displacement in the silanol region were observed. This is consistent with physical adsorption of the formulation onto the carrier rather than chemical interaction. However, the interaction between olmesartan medoxomil and the Syloid carrier cannot be assessed by FTIR. This is because the drug represents only about 1% *w*/*w* of the adsorbed system after solidification. Therefore, DSC and XRD were performed to establish the physical state of the drug after adsorption.

**Figure 7:**
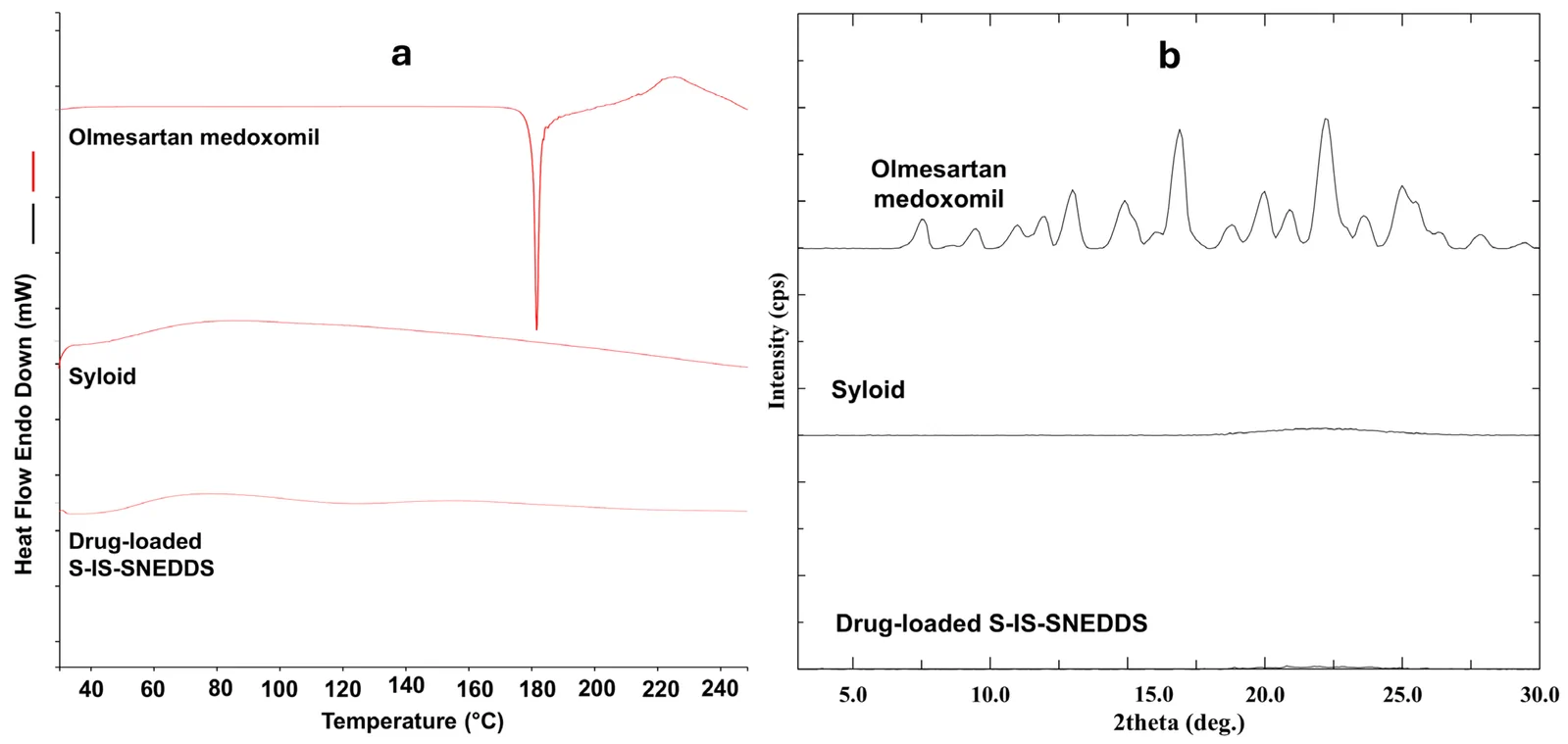
(**a**) DSC thermograms and (**b**) X-ray diffraction patterns of raw olmesartan medoxomil, Syloid, and the drug-loaded S-IS-SNEDDS. Traces are offset vertically for clarity.

The physical state of the drug in the adsorbed system was examined by differential scanning calorimetry and X-ray diffraction (Figure 7). Raw olmesartan medoxomil melted sharply at 181.7 °C, which is consistent with a previously reported study [24]. On the other hand, drug-loaded S-IS-SNEDDS showed a similar flat thermogram compared to Syloid. This suggests that olmesartan medoxomil is present in an amorphous state within the formulation pores. For confirmatory assessment, XRD was performed, and raw olmesartan medoxomil showed numerous sharp reflections, with two intense peaks at 22.2° and 16.9° 2θ. In alignment with DSC, the absence of diffraction peaks in drug-loaded S-IS-SNEDDS indicates drug amorphization. Both techniques indicate that the drug remains molecularly dissolved within the adsorbed vehicle rather than crystallizing on the carrier.

### 3.7. Evaluation of Matrix-Based SNEDDS

To overcome the limitations of the adsorption-based solid form, the L-IS-SNEDDS was instead incorporated into two thermoresponsive matrices. These matrices stay solid during storage and revert to a liquid state at body temperature. This was inspired by the inverted behavior of the poloxamer polymer in nonaqueous solvents [16]. This inverted thermoresponsive behavior could enable the entire SNEDDS to be released without the entrapment drawback. This was achieved by combining poloxamer with two nonaqueous solvents (propylene glycol and polyethylene glycol 400). The mechanistic transition of the inverted thermoresponsive poloxamer was investigated using Fourier transform infrared (FTIR) spectroscopy, DSC, and XRD. This allowed elucidation of the chemical interactions between the agents used and the physical state of poloxamer within the matrix.

#### 3.7.1. FTIR Spectroscopy of the Binary Matrices

**Figure 8:**
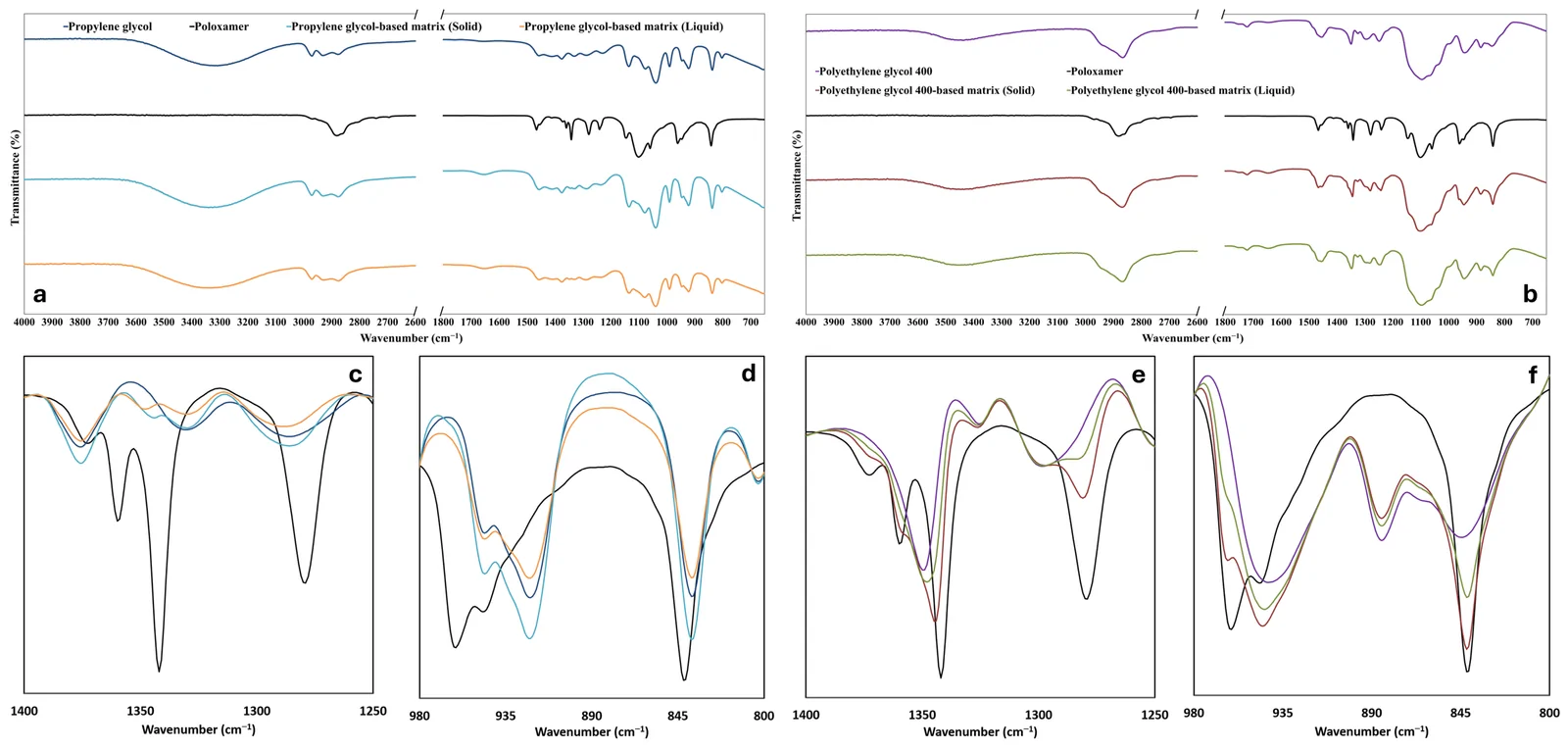
FTIR spectra of the binary poloxamer/solvent matrices. (**a**) Propylene glycol, poloxamer and the propylene glycol-based matrix in the solid and liquid states. (**b**) Polyethylene glycol 400, poloxamer, and the polyethylene glycol 400-based matrix in the solid and liquid states. (**c**,**d**) Expansions of the CH_2_ wagging and twisting region (1400–1250 cm^−1^) and the CH_2_ rocking region (980–800 cm^−1^) for the propylene glycol-based system. (**e**,**f**) The corresponding regions for the polyethylene glycol 400-based system.

FTIR spectra of pure poloxamer, propylene glycol, polyethylene glycol 400, and the respective formulations in both solid and liquid states are presented in Figure 8. The FTIR spectrum of pure poloxamer exhibited characteristic peaks of the poly(ethylene oxide)–poly(propylene oxide)–poly(ethylene oxide) (PEO–PPO–PEO) triblock structure. Among them, the CH_2_ wagging doublet at 1342–1344 and 1360 cm^−1^ and the CH_2_ twisting band at 1279 cm^−1^ are diagnostic markers of the crystalline PEO blocks in their ordered helical conformation [47,48,49]. These bands are sensitive to the crystalline-to-amorphous transition. An additional band at 961 cm^−1^, assigned to CH_2_ rocking, was used to monitor the solid-to-liquid transition of the matrix [47,50].

In the poloxamer/propylene glycol System, the CH_2_ wagging/twisting and CH_2_ rocking regions were selected to investigate the thermal transition mechanism. In this system, the crystalline PEO markers of poloxamer were of low intensity in both physical states. The CH_2_ wagging band at 1342–1344 cm^−1^ and the CH_2_ twisting band at 1279 cm^−1^ were only faintly observed. This is ascribed to the partial overlap of the vibrational modes of propylene glycol at 1335 and 1287 cm^−1^ [51]. However, both were more intense in the solid state than in the liquid state. On the other hand, the 1360 cm^−1^ component of the wagging doublet could not be resolved. In the rocking region, the 961 cm^−1^ component was likewise weak in both states. Poloxamer markers were slightly more intense in the solid (cooled) state than in the liquid (heated) state. This may reflect loss of residual crystalline order upon melting. However, the weakness of these markers, even in the solid state, suggests that propylene glycol extensively disrupts the ordered PEO helices prior to the thermal transition [47]. This leaves little crystalline order to be lost upon heating. This behavior is consistent with propylene glycol’s role as a small-molecule diol that intercalates between poloxamer chains and disrupts their helical packing.

In the poloxamer/polyethylene glycol 400 system, the crystalline PEO markers were well resolved and showed pronounced changes between the two states. The CH_2_ wagging band at 1342–1344 cm^−1^ and the CH_2_ twisting band at 1279 cm^−1^ were intense in the solid (cooled) state. Both were markedly attenuated in the liquid (heated) state. In addition, the 1360 cm^−1^ component of the wagging doublet and the 961 cm^−1^ rocking band were each resolved as a distinct shoulder in the solid state. Both diminished substantially upon melting. Because polyethylene glycol 400 contributes overlapping bands in these regions, we evaluated the poloxamer-specific contribution relative to the pure polyethylene glycol 400 spectrum [52]. On this basis, the transition of the PEO blocks from an ordered helical to a disordered amorphous state could be expected [47,48]. The retention of intense crystalline markers in the solid state indicates that poloxamer preserves substantial PEO crystallinity within the polyethylene glycol 400 matrix. This could be ascribed to the structural identity between polyethylene glycol 400 and the PEO blocks of poloxamer. Both share the same –CH_2_CH_2_O– repeat unit, which provides a compatible environment that could template the helical packing [47,48,50]. In contrast to propylene glycol, polyethylene glycol 400 stabilizes the crystalline order in the solid state and releases it only upon heating to the transition temperature.

#### 3.7.2. DSC of the Binary Matrices

**Figure 9:**
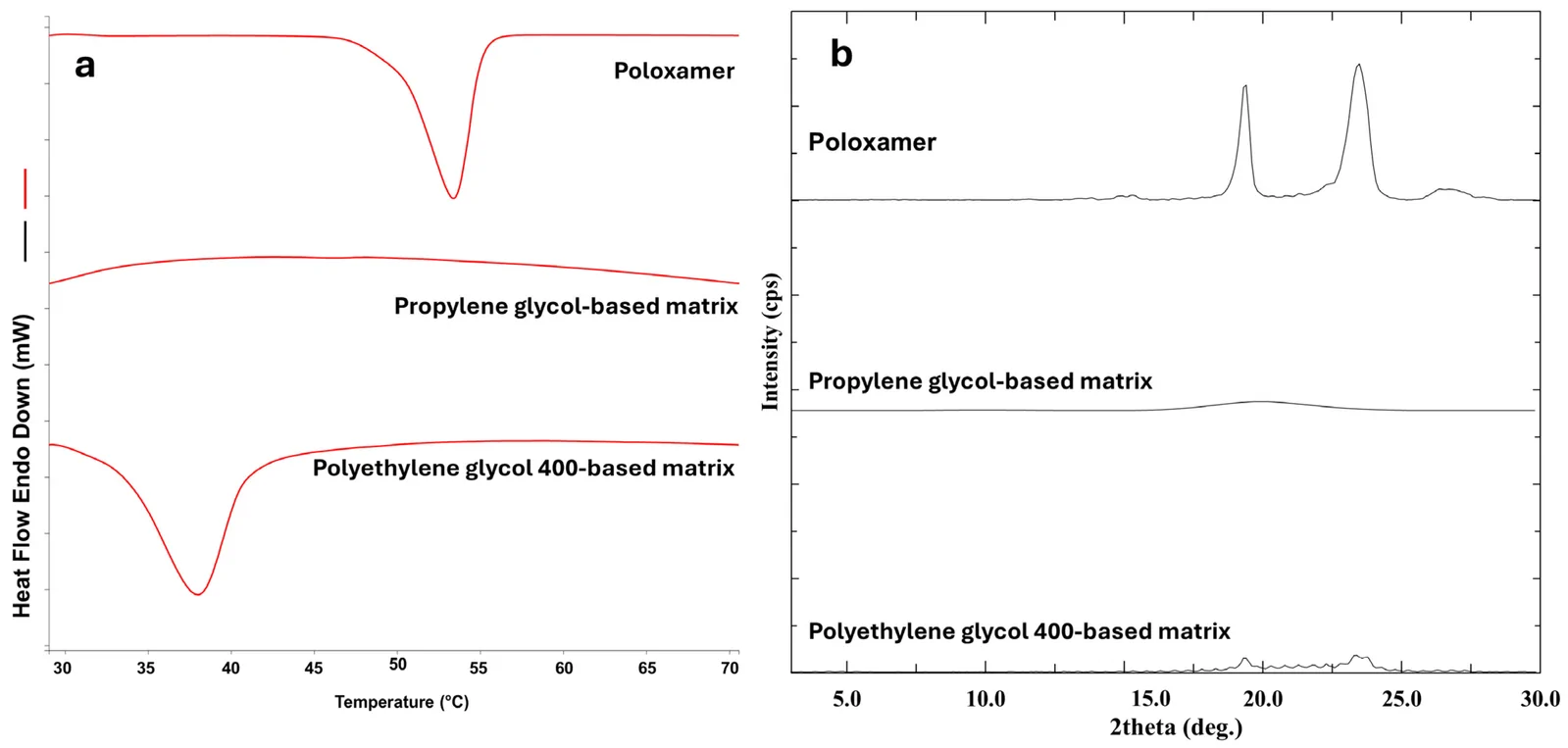
(**a**) DSC thermograms and (**b**) X-ray diffraction patterns of poloxamer, propylene glycol-based matrix, and polyethylene glycol 400-based matrix.

DSC was used to examine the thermal performance of the two matrix types (Figure 9). The results obtained showed that poloxamer 188 melted at 53.4 °C. The polyethylene glycol 400-based matrix retained a melting endotherm of poloxamer 188 at 38.2 °C. In contrast, the thermogram of the propylene glycol-based matrix showed no transition. This finding agrees with FTIR results, which showed polyethylene glycol 400 preserved most of the crystalline order of the poly(ethylene oxide) blocks. However, the observed depression of the poloxamer melting temperature could be ascribed to the plasticizing effect of polyethylene glycol 400. For further assessment, XRD was performed to provide clear insight into poloxamer crystallinity in both matrices.

#### 3.7.3. XRD of the Binary Matrices

X-ray diffraction (Figure 9) was used to examine the crystalline order of the matrices directly. Pure poloxamer 188 showed two sharp reflections characteristic of crystalline poly(ethylene oxide) at 19.5° and 23.6° 2θ. Both peaks were retained in the polyethylene glycol 400-based matrix, at 19.4° and 23.4° 2θ. The observed intensity reduction reflects its concentration in the matrix. In contrast, the X-ray diffraction patterns of the propylene glycol-based matrix showed no sign of crystallinity. This was indicated by the absence of characteristic sharp poloxamer peaks. The observed difference between the two solvents agreed with FTIR and DSC results. This could be attributable to their structural relationship to the polymer. Although neither DSC nor XRD detected crystalline poloxamer in the propylene glycol-based matrix, crystalline FTIR markers were still observed in the solid state and diminished upon heating. Moreover, the observed reverse visual transition of the propylene glycol-based matrix from solid to liquid state supports this conclusion. Therefore, the discrepancy reflects the differing sensitivity of the three techniques rather than the true absence of crystallinity of poloxamer in the propylene glycol-based matrix. Conversely, vibrational spectroscopy remains sensitive to small or poorly ordered crystalline domains within the matrix system.

### 3.8. Pharmaceutical Assessment of T-IS-SNEDDS Formulations

**Figure 10:**
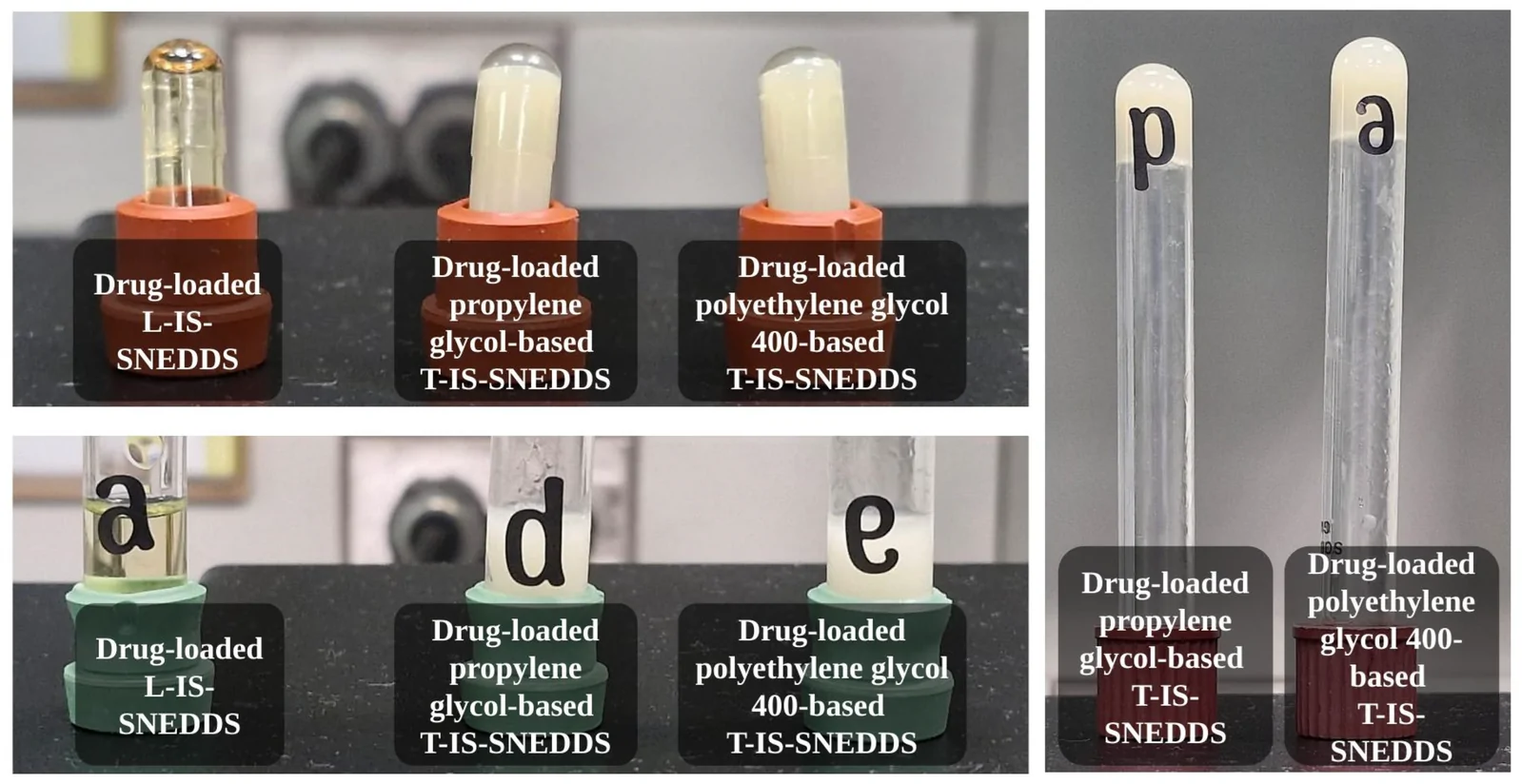
Capsules filled with the drug-loaded L-IS-SNEDDS, the drug-loaded propylene glycol-based T-IS-SNEDDS, and the drug-loaded polyethylene glycol 400-based T-IS-SNEDDS (**top**), and the volume occupied by the same formulations in test tubes (**bottom**). The inversion of tubes containing drug-loaded propylene glycol-based and polyethylene glycol 400-based T-IS-SNEDDS is shown to demonstrate shape retention without flow (**right**).

In contrast to the adsorption approach, combining L-IS-SNEDDS with thermoresponsive matrices did not adversely affect dosage volume (Figure 10). Both matrix-based formulations occupied a volume close to that of the parent liquid formulation. This is because the matrix formers dissolve within the formulation rather than acting as a bulky solid adsorbent. Accordingly, the therapeutic dose fit within a single capsule. This represents a fourfold reduction in the number of capsules relative to the S-IS-SNEDDS formulation. Moreover, both formulations retained their shape upon inversion at ambient temperature, and no flow was observed (Figure 10). This confirms that the prepared systems exist as non-flowing solids under storage and handling conditions. This addresses the leakage and capsule-incompatibility limitations associated with the liquid form. Therefore, the thermoresponsive matrix combines the volume efficiency of the liquid formulation with the physical stability required of a solid dosage form.

**Figure 11:**
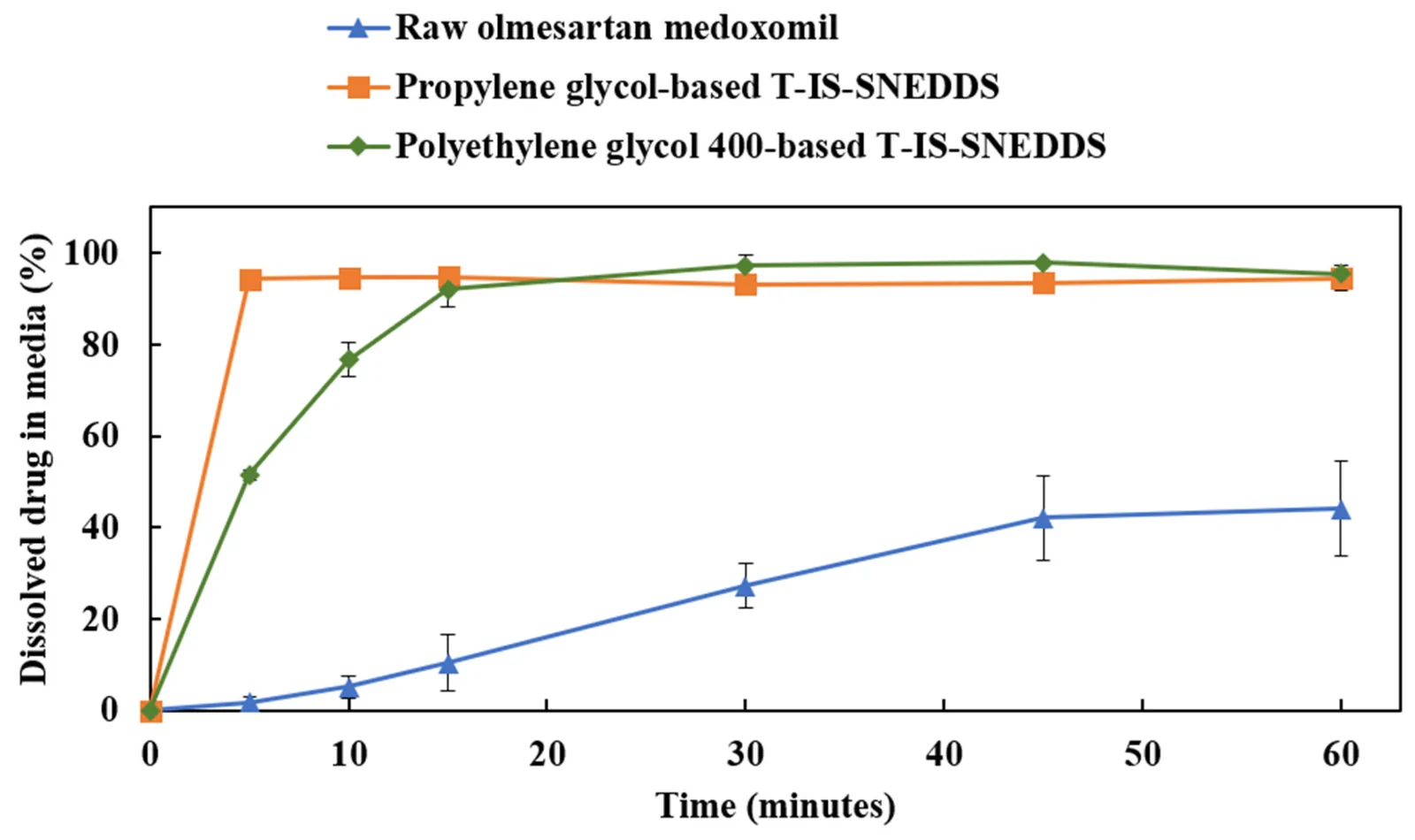
In vitro dissolution of raw olmesartan medoxomil and the drug-loaded propylene glycol-based and polyethylene glycol 400-based T-IS-SNEDDS.

The self-emulsifying ability after solidification of both T-IS-SNEDDS formulations was assessed. The propylene glycol-based system dispersed into droplets of 284.6 ± 1.5 nm with a polydispersity index of 0.535 ± 0.041. Furthermore, the polyethylene glycol 400-based system produced nanoemulsion droplets (180.1 ± 4.6 nm) with a polydispersity index of 0.471 ± 0.053. This finding indicates that incorporating the L-IS-SNEDDS into the thermoresponsive matrices did not negatively affect the formulation’s emulsification tendency. For further assessment, we studied the in vitro dissolution profiles of both systems to investigate their impact on olmesartan medoxomil dissolution. Figure 11 presents the in vitro dissolution profiles of raw olmesartan medoxomil and the T-IS-SNEDDS formulations. Both matrix systems markedly enhanced olmesartan medoxomil dissolution. The current results showed 94.5 ± 2.8% and 95.5 ± 1.8% olmesartan medoxomil dissolved at 60 min for the propylene glycol-based and polyethylene glycol 400-based systems, respectively. In addition, both formulations achieved high dissolution efficiency (DE = 90.1% and 86.8%, respectively). The observed ≈95% release of olmesartan medoxomil within 60 min from both matrix systems overcame the incomplete release observed with the solid adsorbent form.

The current results revealed that the two matrix systems differed primarily in their initial dissolution rates. The propylene glycol-based formulation exhibited immediate release, with 94.4 ± 0.9%, 94.7 ± 0.9%, and 94.8 ± 0.8% dissolved at 5, 10, and 15 min. In contrast, the polyethylene glycol 400-based formulation showed a slower dissolution rate. It dissolved 51.6 ± 1.1%, 76.7 ± 3.7%, and 92.1 ± 3.9% over the same intervals. This corresponds to an initial dissolution rate of 18.9%/min for the propylene glycol-based system, compared with 10.3%/min for the polyethylene glycol 400-based system. The slower initial dissolution rate of the polyethylene glycol 400-based formulation could be attributed to the stronger matrix formed by the longer polyethylene glycol 400 chains. This agrees with FTIR, DSC, and XRD results of the binary matrices (Section 3.7). The structural identity between the PEO blocks of poloxamer and the longer polyethylene glycol 400 molecule preserves partial crystalline ordering and produces a more rigid matrix [47,48,50]. In contrast, the shorter propylene glycol may bridge adjacent chains and facilitate conversion of the matrix to a liquid state.

### 3.9. Characterization of the Prepared T-IS-SNEDDS Formulations

#### 3.9.1. FTIR Spectroscopy of the Drug-Loaded T-IS-SNEDDS

**Figure 12:**
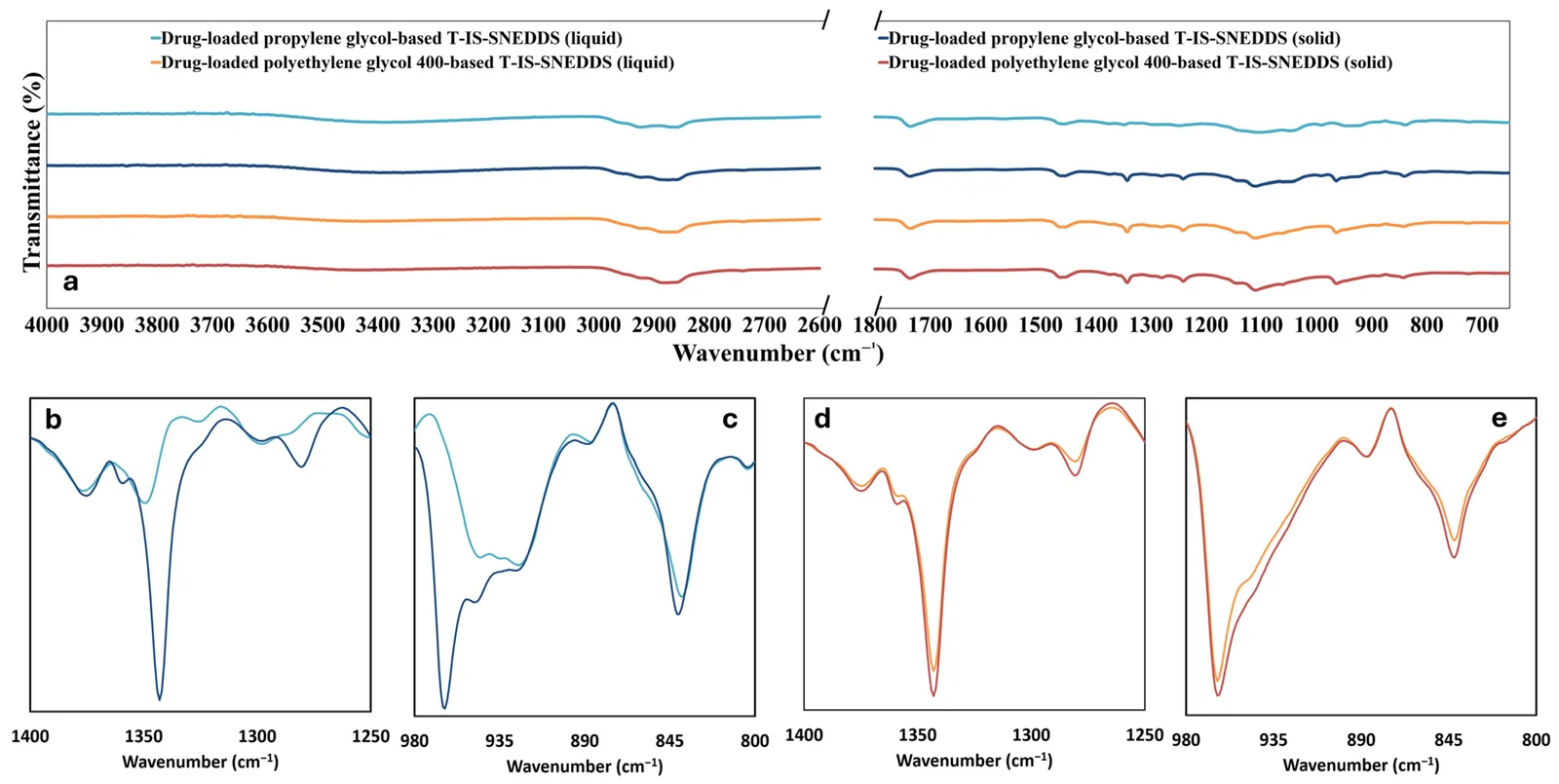
FTIR spectra of the drug-loaded T-IS-SNEDDS formulations in the solid and liquid states. (**a**) Full spectra of the propylene glycol-based and polyethylene glycol 400-based formulations in both states. (**b**,**c**) Expansions of the CH_2_ wagging and twisting region (1400–1250 cm^−1^) and the CH_2_ rocking region (980–800 cm^−1^) for the propylene glycol-based formulation. (**d**,**e**) The corresponding regions for the polyethylene glycol 400-based formulation.

The spectra of the drug-loaded T-IS-SNEDDS formulations in the solid and liquid states are presented in Figure 12. In the solid state, both formulations retained the crystalline PEO markers of the poloxamer. This indicates that the polymer crystallizes within the L-IS-SNEDDS formulation as it does in the matrix system. In the propylene glycol-based formulation, the 1343, 1281, and 961 cm^−1^ markers were markedly attenuated in the liquid state (Figure 12b,c). In contrast, the polyethylene glycol 400-based formulation showed only modest attenuation (Figure 12d,e). This is consistent with the overlapping contributions of amorphous polyethylene glycol 400 and polysorbate ethoxylate chains in these regions. Overall, these observations indicate loss of crystalline order upon melting. Therefore, DSC and XRD were used to study the impact of the L-IS-SNEDDS formulation on poloxamer crystallinity.

#### 3.9.2. DSC of the Drug-Loaded T-IS-SNEDDS

**Figure 13:**
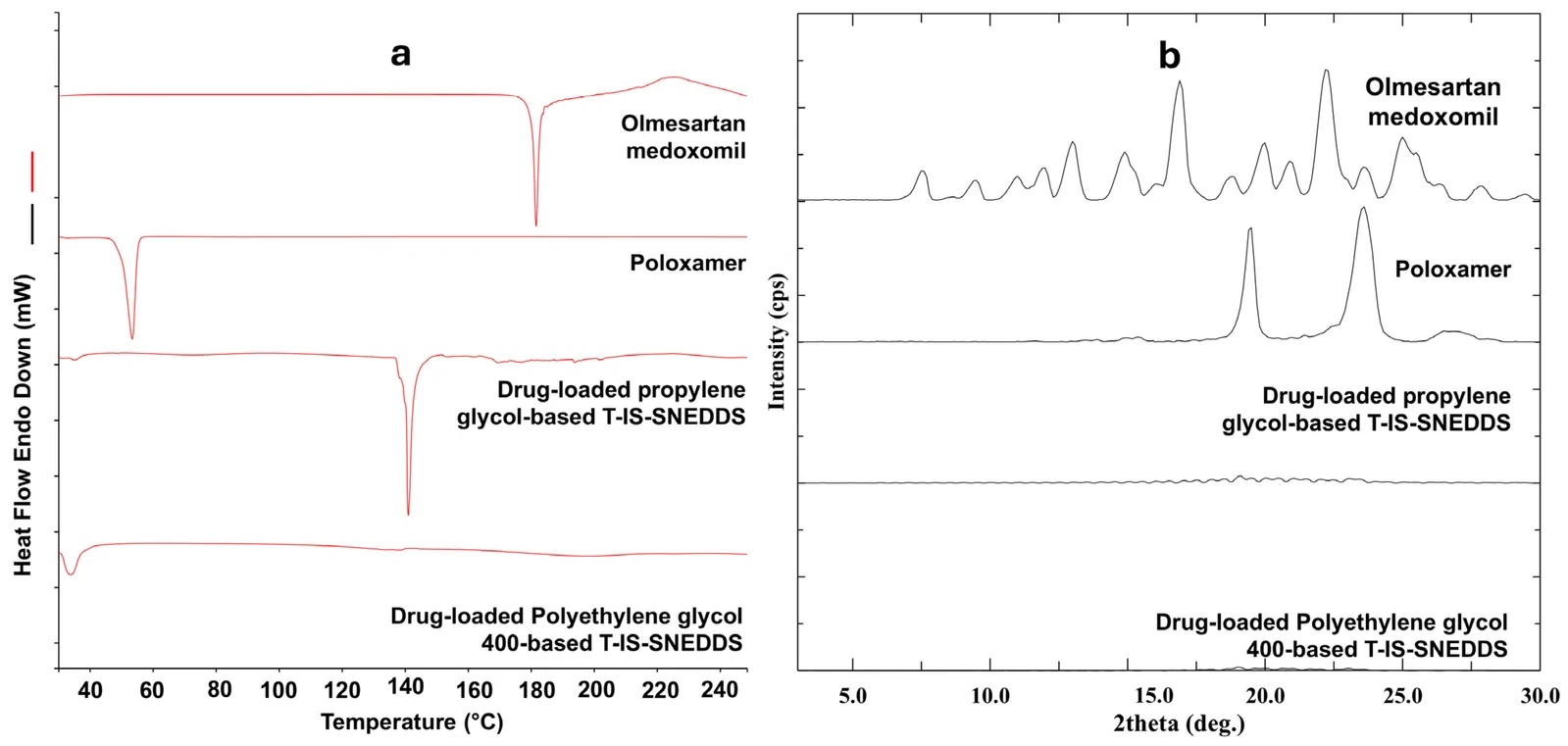
(**a**) DSC thermograms and (**b**) X-ray diffraction patterns for raw olmesartan medoxomil, poloxamer 188, the drug-loaded propylene glycol-based T-IS-SNEDDS, and the drug-loaded polyethylene glycol 400-based T-IS-SNEDDS.

Figure 13 shows the thermograms of the drug-loaded formulations against poloxamer and raw olmesartan medoxomil. Both systems showed a melting endotherm in the matrix-transition region, at 36.2 °C for the propylene glycol-based formulation and 34.1 °C for the polyethylene glycol 400-based formulation. This confirms that both formulations liquefy below body temperature while remaining solid at ambient storage temperature. The result for the propylene glycol-based system is particularly interesting. This is because the corresponding matrix system showed no detectable transition. However, the appearance of a melting endotherm once the self-nanoemulsifying components are incorporated indicates that these components dilute the propylene glycol. Consequently, this permits partial recrystallization of the PEO blocks of poloxamer. This finding suggests that propylene glycol suppresses crystallinity in a concentration-dependent manner rather than eliminating it completely. Moreover, the observed crystalline state could be attributed to other components in the SNEDDS formulation, such as Tween 80. However, neither formulation showed a melting endotherm attributable to olmesartan medoxomil. This indicates that the drug could remain molecularly dispersed within the matrix in an amorphous state. A further sharp endothermic event was recorded at 141.5 °C in the propylene glycol-based formulation. This is ascribed to the volatile nature of the propylene glycol solvent.

#### 3.9.3. XRD of the Drug-Loaded T-IS-SNEDDS

Figure 13 shows the diffraction patterns of the drug-loaded formulations. Even though the poloxamer content (20% *w*/*w*) is the same as in the binary matrices, both poloxamer diffraction peaks were absent in both T-IS-SNEDDS formulations. This could be ascribed to a lower degree of crystallinity and less ordered PEO crystallites in the presence of the L-IS-SNEDDS components. Nevertheless, this does not prove poloxamer amorphization in either formulation. Both calorimetry and infrared spectroscopy detected crystalline poloxamer in the same formulations. Therefore, diffraction was the least sensitive of the three techniques used.

## 4. Conclusions

This study demonstrates that combining in situ salt formation with a thermoresponsive poloxamer matrix overcomes the two principal limitations of adsorption-based solid SNEDDS for the weakly acidic, poorly water-soluble drug olmesartan medoxomil. Excipient screening identified Imwitor 308 and Tween 80 as the optimal oil and surfactant, and sodium carbonate increased drug solubility from 4.11 to 46.01 mg/g through in situ salt formation, whereas calcium carbonate was ineffective because it is insoluble in the anhydrous vehicle. Conventional adsorption onto Syloid required four capsules and released only 67.76 ± 3.18% of the dose, reflecting entrapment of the formulation within the mesoporous carrier. In contrast, both poloxamer-based thermoresponsive matrices accommodated the therapeutic dose in a single capsule and achieved complete dissolution (≈95%), with the propylene glycol matrix releasing the drug faster than the polyethylene glycol 400 matrix. FTIR analysis attributed this difference to the greater degree of preserved PEO crystallinity in the polyethylene glycol 400 system. Collectively, the thermoresponsive in situ salified SNEDDS (T-IS-SNEDDS) platform offers a practical single-unit solid formulation strategy for weakly acidic, poorly water-soluble drugs. Further in vivo pharmacokinetic and stability studies are warranted to confirm the in vitro advantages reported here.

## Figures and Tables

**Table 1:** Olmesartan medoxomil solubility in oils.

Oil	Olmesartan Medoxomil Solubility (mg/g)
Oleic acid	0.08 ± 0.004
Imwitor 308	1.23 ± 0.078
Peceol	0.41 ± 0.019
Maisine 35-1	0.28 ± 0.015
Miglyol 810N	0.07 ± 0.003
Captex 355	0.06 ± 0.001
Soybean oil	0.06 ± 0.002
Arachis oil	0.08 ± 0.003

Data are presented as mean ± SD (*n* = 3).

**Table 2:** Olmesartan medoxomil solubility in SNEDDS formulations.

Surfactant Type in Formulation	Olmesartan Medoxomil Solubility (mg/g)
Tween 85	2.05 ± 0.13
Tween 80	4.11 ± 0.11
Tween 60	2.46 ± 0.02
Tween 20	2.73 ± 0.07
HCO-60	2.68 ± 0.19
HCO-30	2.30 ± 0.04
HCO-10	1.34 ± 0.07

Data are presented as mean ± SD (*n* = 3).

**Table 3:** Solubility of olmesartan medoxomil in the SNEDDS modified with sodium carbonate or calcium carbonate.

Concentration (mg/g)	Olmesartan Medoxomil Solubility (mg/g, Mean ± SD)	
	Sodium Carbonate (Na_2_CO_3_)	Calcium Carbonate (CaCO_3_)
0	4.11 ± 0.11	
2	17.78 ± 0.62	5.59 ± 0.11
4	26.77 ± 0.67	5.60 ± 0.11
6	32.74 ± 0.25	5.79 ± 0.16
8	39.08 ± 2.08	5.71 ± 0.16
10	46.01 ± 1.92	5.68 ± 0.13

Data are presented as mean ± SD (*n* = 3).

## Data Availability

The original contributions presented in this study are included in the article. Further inquiries can be directed to the corresponding author.
